# Extracellular Vesicles Derived From Adult and Fetal Bone Marrow Mesenchymal Stromal Cells Differentially Promote *ex vivo* Expansion of Hematopoietic Stem and Progenitor Cells

**DOI:** 10.3389/fbioe.2021.640419

**Published:** 2021-02-25

**Authors:** Corina A. Ghebes, Jess Morhayim, Marion Kleijer, Merve Koroglu, Stefan J. Erkeland, Remco Hoogenboezem, Eric Bindels, Floris P. J. van Alphen, Maartje van den Biggelaar, Martijn A. Nolte, Bram C. J. van der Eerden, Eric Braakman, Carlijn Voermans, Jeroen van de Peppel

**Affiliations:** ^1^Department of Hematopoiesis, Sanquin Research, Amsterdam, Netherlands; ^2^Department of Hematology, Erasmus MC, University Medical Center, Rotterdam, Netherlands; ^3^Department of Immunology, Erasmus MC, University Medical Center, Rotterdam, Netherlands; ^4^Department of Molecular Hematology, Sanquin Research, Amsterdam, Netherlands; ^5^Department of Internal Medicine, Erasmus MC, University Medical Center, Rotterdam, Netherlands

**Keywords:** extracellular vesicles, BMSCs, HSPC expansion, transplantation, hematopoietic niche, intercellular communication, EV cargo

## Abstract

Recently, we and others have illustrated that extracellular vesicles (EVs) have the potential to support hematopoietic stem and progenitor cell (HSPC) expansion; however, the mechanism and processes responsible for the intercellular communication by EVs are still unknown. In the current study, we investigate whether primary human bone marrow derived mesenchymal stromal cells (BMSC) EVs isolated from two different origins, fetal (fEV) and adult (aEV) tissue, can increase the relative low number of HSPCs found in umbilical cord blood (UCB) and which EV-derived components are responsible for *ex vivo* HSPC expansion. Interestingly, aEVs and to a lesser extent fEVs, showed supportive *ex vivo* expansion capacity of UCB-HSPCs. Taking advantage of the two BMSC sources with different supportive effects, we analyzed the EV cargo and investigated how gene expression is modulated in HSPCs after incubation with aEVs and fEVs. Proteomics analyses of the protein cargo composition of the supportive aEV vs. the less-supportive fEV identified 90% of the Top100 exosome proteins present in the ExoCarta database. Gene Ontology (GO) analyses illustrated that the proteins overrepresented in aEVs were annotated to oxidation-reduction process, mitochondrial ATP synthesis coupled proton transport, or protein folding. In contrast, the proteins overrepresented in fEVs were annotated to extracellular matrix organization positive regulation of cell migration or transforming growth factor beta receptor (TGFBR) signaling pathway. Small RNA sequencing identified different molecular signatures between aEVs and fEVs. Interestingly, the microRNA cluster miR-99b/let-7e/miR-125a, previously identified to increase the number of HSPCs by targeting multiple pro-apoptotic genes, was highly and significantly enriched in aEVs. Although we identified significant differences in the supportive effects of aEVs and fEVs, RNAseq analyses of the 24 h treated HSPCs indicated that a limited set of genes was differentially regulated when compared to cells that were treated with cytokines only. Together, our study provides novel insights into the complex biological role of EVs and illustrates that aEVs and fEVs differentially support *ex vivo* expansion capacity of UCB-HSPCs. Together opening new means for the application of EVs in the discovery of therapeutics for more efficient *ex vivo* HSPC expansion.

## Introduction

Allogeneic hematopoietic stem cell transplantation (HSCT) has become a common practice for the treatment of (malignant-) hematopoietic diseases (Copelan, 2006; Juric et al., 2016). However, most patients in need for HSCT do not have a suitable human leukocyte antigen (HLA)-matched related donor, and of these, less than half can find an HLA-matched unrelated donor (Gragert et al., 2014). For these patients, umbilical cord blood (UCB) has become an important hematopoietic stem and progenitor cell (HSPC) source for allogeneic HSCT. In contrast to peripheral blood stem cell transplantation (PBSCT), UCB has fewer mature T lymphocytes, thus allowing UCB transplantation with a greater degree of HLA mismatch (Stanevsky et al., 2009), and the large number of banked UCB units can easily facilitate the finding of an HLA-matched graft (Mayani et al., 2020). However, the relatively low number of HSPCs present in one UCB unit is a major limitation for UCB transplantation (de Lima et al., 2012; Kindwall-Keller and Ballen, 2020). This is associated with delayed engraftment and higher risk of graft failure, and leads to restriction in their widespread application (Mattsson et al., 2008). To overcome these limitations, the development of efficient culture conditions and the discovery of new compounds that boost *ex vivo* HSPC expansion will therefore help toward the treatment of malignant hematopoietic diseases.

In the bone marrow microenvironment, HSPCs are supported by a large heterogeneous population of stromal cells in the perivascular niche, such as endothelial and mesenchymal cells that generate signals regulating hematopoietic stem cells (HSC) self-renewal, quiescence and differentiation (Boulais and Frenette, 2015; Crane et al., 2017). We and others have shown that *ex vivo* co-culture of UCB-CD34^+^ cells with bone marrow-derived mesenchymal stromal cells (BMSCs) increases the number of HSPCs regardless of the presence of growth factors, such as SCF, Flt3L, TPO, IL6, and G-CSF, making BMSCs an exceptional tool to identify novel HSPC regulators (de Lima et al., 2012; Paciejewska et al., 2016; Cooney, 2018). Recent findings suggest that BMSC-derived extracellular vesicles (EVs) may play an important role in the biological functions of their parental cells (Vonk et al., 2018; Harrell et al., 2019). We therefore postulate that EVs derived from BMSCs may recapitulate the hematopoietic supportive effects of their parental cells and can help us to identify new molecules capable of *ex vivo* expansion of relevant UCB-HSPC numbers for the treatment of patients in need for stem cell transplantation.

The HSPCs in the fetal and adult bone marrow niche actively expand, however, the cellular and extracellular compartment within the fetal- and adult bone marrow differ a lot (Gao et al., 2018). Here we aim to assess whether the EVs released by primary human BMSCs of different origins, e.g., fetal and adult BMSCs, have similar differential supportive effects as their parental cells (Paciejewska et al., 2016) and identify the EVs cargo molecules that mediate the HSPC-supporting capacity of stromal cells. To achieve this, we identified the EV cargo molecules by performing proteomics and small non-coding RNA analyses, and studied their effects on the gene expression of UCB-CD34^+^ cells by next generation sequencing. This study identified new regulatory proteins for the application of EVs in the discovery of therapeutics for more efficient *ex vivo* HSPC expansion.

## Materials and Methods

### Isolation and Culture of Human Bone Marrow Derived MSCs

Adult human bone marrow aspirates (40–70 years old) were obtained from the sternum of patients undergoing cardiac surgery after given informed consent and approval of the medical ethics review board of the AMC (MEC:04/042#04.17.370). Collection of fetal tissues for research purposes was approved by the medical ethical review board of the Academic Medical Centre (AMC) (MEC: 03/038). The fetal human bone marrow samples were obtained from the HIS facility of the AMC, Amsterdam. All material has been collected from donors from whom a written informed consent for the use of the material for research purposes had been obtained by the Bloemenhove clinic (Heemstede, The Netherlands). These informed consents are kept together with the medical record of the donor by the clinic. Low-density mononuclear cells (MNC) were separated by Ficoll gradient centrifugation and cultured in Dulbecco’s modified Eagle’s medium, GlutaMAX^TM^ and low glucose (Thermo Fisher Scientific, United States) supplemented with 10% fetal bovine serum (FBS, Bodinco, The Netherlands) and 1% penicillin-streptomycin (Sigma Aldrich, Germany), referred in this article as BMSC medium. Non-adherent cells were removed by replacing the medium after 48 h of incubation. Cells were either frozen or expanded up to passage 5, in order to harvest sufficient amounts of EVs.

### Isolation and Culture of Human UCB-CD34^+^ Cells

UCB was collected after informed consent, according to the guidelines of NetCord FACT (by the Sanquin Cord Blood bank, The Netherlands). CD34^+^ cells were isolated by magnetic cell sorting (MACS, Miltenyi Biotec, Germany), using the human CD34^+^ Microbead kit (Miltenyi; 130-046-703, Germany) according to manufacturer instructions and within 48 h after initial sample collection. This resulted in a purity of more than 90% CD34^+^ cells, as determined by flow cytometry and were immediately frozen. After thawing of frozen cells, 10,000 viable UCB-CD34^+^ CD45^+^ cells, referred here as UCB-CD34^+^, were cultured in 48 well plates in growth factor driven serum-free expansion media, Cellgenix GMP SCGM (Cellgenix, Germany) supplemented with stem cell factor (recombinant human SCF, 50 ng/mL, Biolegend, United States) and FMS-like tyrosine kinase 3 ligand (Flt3L, 50 ng/mL, Prepotech, United Kingdom), with or without EVs. Cells were refreshed every 2–3 days. After 10 days the total number of cells (TNCs), CD34^+^ cells and primitive HSC was counted. TNC number was obtained using fluorescence counting beads in combination with DAPI (diamidino-2-phenylindole, Sigma Aldrich, Germany) for live/dead staining. For the TGFB1 receptor inhibitor experiment, cells were incubated in the absence or presence of fEVs, 1 μM TGFB1 receptor inhibitor (LY 2157299 from Axon Medchem, Netherlands) and DMSO (control TGFB1R inhibitor).

#### Colony Forming Unit (CFU) Assay

UCB-CD34^+^ cells, non-cultured and cultured for 10 days, were plated in a 24-well plate at 150 cell/well in cytokine-supplemented methylcellulose medium (MethoCult H4435, StemCell Technologies, Canada) and further cultured for 14 days at 37°C and 5% CO_2_. Colony forming unit colonies were counted using an inverted bright field microscope (Leica, Germany).

### Isolation and Characterization of Human MSC Derived EVs

Adult and Fetal BMSC-derived EVs were isolated from BMSCs supernatant, after confluent layers of cells cultured in T175 flasks were exposed for 24 h to serum-free BMSC medium. The conditioned BMSC supernatant was exposed to low speed centrifugation (300 g for 5 min, 2,000 g for 10 min) followed by ultracentrifugation (20,000 g for 30 min and 100,000 g for 1 h) using the Ti50.2 rotor (Beckman Coulter, United States). The EV pellets, further referred in this article as EVs, were collected from the last ultracentrifugation step after the supernatant containing no EV was removed, in expansion media or PBS dependent on the assay to follow.

#### Concentration and Size Distribution

Concentration and size distribution of BMSC-derived EVs were determined using NanoSight NS300 (Nanosight Ltd., United Kingdom) equipped with a 405 nm laser. The particles in each sample were recorded for 5 times 60 s. The data was processed by NTA 2.3 software.

##### Flow Cytometry

UCB-CD34^+^ cells, before and after 10 days’ culture, were examined for surface markers and viability. Absolute numbers were determined using fluorescence reference counting beads (Thermo Fisher Scientific, United States), anti-APC-CD34^+^ (Biolegend, United Kingdom) or anti-PeCy7-CD34^+^ (BD Bioscience, United States), anti-FITC-CD45 (BD Bioscience, United States), anti-APC-CD38 (eBioscience, Thermo Fisher Scientific, United States), anti-PE-CD45RA (Diaclone, France), and DAPI (Sigma Aldrich, Germany). All samples were analyzed using LRS II (BD Biosciences, United States) and data was analyzed using FlowJo software (Tree Star, Inc., United States).

#### Transmission Electron Microscopy (TEM)

TEM images were taken by negative staining of the EVs, as previously described (Morhayim et al., 2020). Freshly carbon sputtered and formvar coated copper grids were incubated on EV preparations, washed rapidly in water and contrasted with 3.5% uranyl acetate. Grids were blotted and dried before the analysis using a Tecnai T12 G2 Biotwin at 120 kV.

##### Western Blot Analyses

We compared BMSC supernatant with BMSC-derived EVs and supernatant depleted EVs. For this experiment we have collected the supernatants and EVs from confluent cell layers of five T175 flasks, adult and fetal MSCs each, exposed for 24 h in serum free BMSC medium. For supernatant before centrifugation and supernatant depleted EVs we collected 4 ml and concentrated to a volume of approx. 150 μl using a 3 k molecular weight cut-off amicon ultra centrifugation filter (Merck Millipore, Germany). While the aEVs and fEVs were collected from 30 ml of supernatant following the ultracentrifugation steps and resuspended in ∼150 μl of serum free BMSC medium. Protein samples were prepared by immediately mixing with 4X reducing sample buffer containing β-mercaptoethanol (Sigma Aldrich, Germany). EVs proteins were separated by SDS-PAGE at 100 V and transferred onto a nitrocellulose membrane (Whatman Gmbh, Germany). After incubation with 5% BSA for 1 h, the membrane was incubated with primary antibodies against Annexin A2 (ANXA2; rabbit polyclonal. 1:1,000, Abcam, Cambridge, United Kingdom). Membranes were probed with secondary antibody goat anti-rabbit-HRP (Dako, United States) and films were processed with a Konica Minolta, SRX-101A.

##### RNA Isolation, Next-Generation Sequencing and Bioinformatic Analysis of EV Derived Small RNA

EV pellets were collected for each sample from confluent cell layers of 10×T175 flasks, as previously described (Morhayim et al., 2020). Total EV-RNA was isolated using the TRIzol LS reagent (Thermo Fisher Scientific, United States) according to the manufacturer’s instructions. RNA concentration and size distribution profile were analyzed on an Agilent Bioanalyzer RNA 6000 Pico chip (Thermo Fisher Scientific, United States). Small RNA libraries were prepared with the NEBNext Small RNA library preparation kit (New England Biolabs, United States) according to the manufacturer’s instructions. Finished small RNA libraries were quantified on a Bioanalyzer High Sensitivity DNA chip (Agilent, United States) and subsequently normalized and pooled. Single end 50-bp sequencing was performed on a Miseq (Illumin). Subsequently demultiplexing was done using Illumina’s bcl2fastq program allowing for one mismatch in the barcode. Quality metrics on the resulting FASTQ files were generated using fastqc in combination with multiqc. The illumina universal adapters were removed using trim galore. Subsequently alignment was performed using bowtie1 using very sensitive settings (-l15 –tryhard –all –best –strata) Finally quantification of abundance per feature was performed using in house developed software. During quantification fragments were assigned only once to the best matching feature. The feature set used originate from the DASHR (v2) small RNA database. Total number of reads per small RNA ID were counted. Differential gene expression analysis was done with DESeq2 (Love et al., 2014). All small RNAs with a sum count of 2 and higher in all samples were included in the analysis. The sequencing data are available in the Gene Expression Omnibus (GEO) database repository (GSE165323)^1^. miRPathDB v2.0 was used to categorize the miRNA into GO annotations (Kehl et al., 2020).

##### RNA Isolation and RNA Profiling of UCB-CD34^+^ Cells Transcriptome

Freshly isolated UCB-CD34^+^ cells were immediately frozen or cultured for 24 h in the absence or presence of aEVs or fEVs. Total RNA was isolated using NucleoSpin RNA XS kit (Macherey-Nagel, Germany) according to the manufacturer’s instructions. RNA concentration and size distribution profile were analyzed on an Agilent Bioanalyzer RNA 6000 Pico chip (Thermo Fisher Scientific, United States). The SMARTer version v4 Ultra Low Input RNA kit for sequencing (Clontech) was used to generate cDNA. Subsequent bulk RNAseq libraries were generated with the Truseq nano DNA sample prep kit (illumina) according to manufacturer’s instructions. The resulting libraries were quality checked and sequenced paired-end 100 cycles on a Novaseq6000 instrument (Illumina). The resulting base-calls from sequencing were converted to FASTQ files using Illumina’s bcl2fastq software allowing for one mismatch in the barcode. Quality metrics of the resulting FASTQ files were summarized using fastqc in combination with multiqc. SMARTer adapters and poly-T tails were removed using fqtrim. Pseudo counts per transcript were measured using salmon. The per transcript pseudo counts were summarized to per gene pseudo counts using the R package tximport. After mapping the reads, differential gene expression analysis was done with DESeq2 (Love et al., 2014) using p.adj < 0.05 as a cutoff for differential gene expression. The sequencing data are available in the Gene Expression Omnibus (GEO) database repository with accession number: GSE165921 (see text footnote 1).

##### Mass Spectrometry and Bioinformatics Analysis of Proteins

Tryptic peptides were prepared according to the method described by Kulak et al. (2014) with some adjustments. Briefly, 4–8 × 10^9^ fEVs and 2–6 × 10^9^ aEVs were isolated, and lysed in 2% Sodium deoxycholate lysis buffer (Sigma Aldrich, Germany), 20 mM TCEP (Tris(2-CarboxyEthyl)Phosphine, Thermo Fisher Scientific, United States), 80 mM ChloroAcetamide (Sigma Aldrich, Germany) and 200 mM TRIS-HCl pH 8.0 (Life Technologies, United Kingdom), boiled at 95°C for 5 min and sonicated for 10 cycles of 30 s on/off in a Bioruptor (Diagenode, Belgium). After an overnight digestion with 100 ng Trypsin-LysC (Promega, United States) at room temperature, samples were acidified with 10% trifluoroacetic acid (Thermo Fisher Scientific, United States) and loaded on in-house prepared SDB-RPS STAGEtips (Empore, United States). The tips were washed with ethyl acetate (Sigma Aldrich, Germany) and 0.2% trifluoroacetic acid (Thermo Fisher Scientific, United States) and the peptides were eluted in three fractions by increasing concentrations (100 mM and 150 mM) of ammonium formate (VWR Chemicals, Belgium) or 5% (v/v) ammonium hydroxide (Merck Millipore, Germany) and acetonitrile (40, 60, and 80% v/v) (BioSolve, France). Sample volume was reduced by SpeedVac and supplemented with 2% acetonitrile, 0.1% TFA to a final volume of 10 μl. Three microliter of each sample was injected for MS analysis. Tryptic peptides were separated by nanoscale C18 reverse phase chromatography coupled on line to an Orbitrap Fusion Lumos Tribrid mass spectrometer (Thermo Fisher Scientific, United States) via a nanoelectrospray ion source (Nanospray Flex Ion Source, Thermo Fisher Scientific, United States). All data was acquired with Xcalibur 4.1 software. The raw mass spectrometry files were processed with the MaxQuant computational platform, 1.6.2.10. Proteins and peptides were identified using the Andromeda search engine by querying the human Uniprot database (downloaded Feb 2019). Standard settings with the additional options match between runs, Label Free Quantification (LFQ), and only unique peptides for quantification were selected. The data was filtered for potential contaminants, reverse hits and “only identified by site” using Perseus 1.6.5.0 (Tyanova et al., 2016). The proteins were filtered for 100% valid values in at least one of the experimental groups. Missing values were imputed by normal distribution (width = 0.3, shift = 1.8), assuming these proteins were close to the detection limit. Double-sided *t*-test (FDR 0.05 and S0 of 4) was used to determine significant differences between aEV and fEV. David Bioinformatics Resources 6.8 was used for gene ontology (GO) analysis, using all the proteins identified by the whole EV lysate proteomics experiment as background. ExoCarta top 100 exosome proteins were downloaded to map the percentage of proteins found back in our proteomics experiment (Keerthikumar et al., 2016). The mass spectrometry proteomics data have been deposited to the ProteomeXchange Consortium^2^ via the PRIDE partner repository with the dataset identifier: PXD022851 (Perez-Riverol et al., 2019).

### Statistics

Statistical analyses were performed with Graphpad Prism 8, unless otherwise stated. Significance was calculated using Student’s *t*-test and two-way ANOVA test. Mean values plus or minus standard deviation of the mean are shown. ^∗^*P* < 0.05; ^∗∗^*P* < 0.01; ^∗∗∗^*P* < 0.001.

## Results

### BMSC-Derived EVs Contain Supportive Factors for *ex vivo* Expansion of Human UCB-CD34^+^ Cells

To analyze whether BMSC-derived EVs are able to support UCB-derived CD34^+^ cells *ex vivo*, we expanded BMSCs from adult and fetal bone marrow and harvested EVs from the conditioned medium by a series of ultracentrifugation steps. We quantified the number of EVs that were produced by the BMSCs and added different numbers of EVs (25,000, 50,000, or 100,000) per human UCB-CD34^+^ cell. *Ex vivo* expansion experiments illustrated that 100,000 EVs were able to support the UCB-CD34^+^ cells, whereas adding a lower number of EVs did not significantly increase the total number of viable nucleated cells as compared to control culture (SCF and Flt3L only) after expansion (Supplementary Figure 1A). Next, we investigated if the isolated BMSC-derived EVs were responsible for *ex vivo* expansion of UCB-CD34^+^ cells or whether other secreted factors were present in the EV-depleted supernatant or serum-free MSC medium. Supplementary Figure 1B illustrates that only the isolated EV-fraction consistently contributed to the increase in the number of viable nucleated cells. These results suggest the presence of supporting factors in the BMSC-derived EVs for the *ex vivo* expansion of UCB-CD34^+^ cells.

### UCB-CD34^+^ Cell Support by BMSC-Derived EVs Is Dependent on the Origin of the BMSCs

**FIGURE 1:**
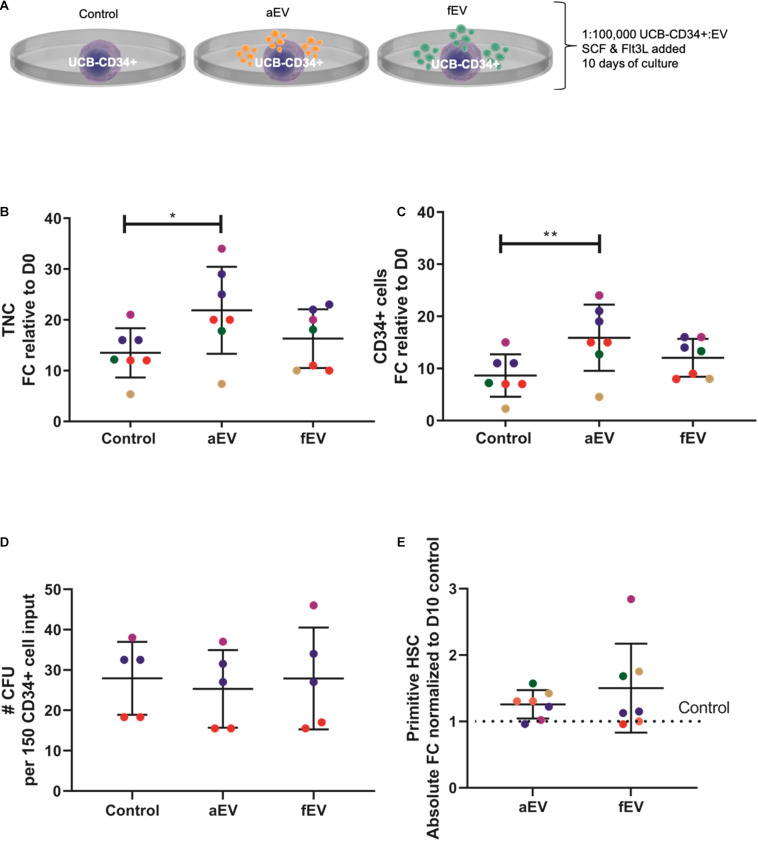
Role of MSC derived EVs in *ex vivo* expansion of UCB-CD34^+^ cells. **(A)** UCB-CD34^+^ cells were cultured for 10 days in growth factor (SCF and Flt3L) driven serum-free expansion media and in the presence of aEVs and fEVs. We compared the effect of single aEVs (*n* = 7) and fEVs (*n* = 7) donors on the expansion of UCB-CD34^+^ cells of the same donor. Each datapoint color represents a different UCB CD34^+^ cell donor, *n* = 5. UCB CD34^+^ cell donors in red and blue were exposed to two different aEV and fEV donors, each. Statistics was performed for all *n* = 7 different aEV and fEV donors. **(B)** Proliferation of viable total nucleated cells, TNC and **(C)** CD34^+^ cells shown as fold change increase relative to Day 0 (D0) input. **(D)** Total number of colony forming cells after being cultured for 10 days in growth factor-driven serum-free expansion media and in the presence of aEVs and fEVs. **(E)** Maintenance of primitive HSC (CD34^+^ CD38-CD45RA-) subset, shown as absolute fold change normalized to D10 cell culture control. *P < 0.05, **P < 0.01.

Next, we compared the supportive effects of BMSC-derived EVs isolated from different origins. UCB-CD34^+^ cells from a single donor were exposed to EVs isolated from adult (aEVs) or fetal (fEVs) BMSCs in growth factor- (SCF and Flt3L) driven serum-free expansion media (Figure 1A). aEVs caused a significant increase in the total number of viable nucleated cells (1.6 fold, *p* < 0.05) and CD34^+^ cell subset (1.8-fold, *p* < 0.01), while fEVs caused a non-significant increase of 1.2 and 1.4 fold, respectively (Figures 1B,C). The expanded UCB-CD34^+^ cells were further examined for their *in vitro* colony forming capacity by performing Colony Forming Unit Granulocyte-Macrophage (CFU-GM) assay. Corrected for input cell numbers, we observed that UCB-CD34^+^ cells treated with either aEVs or fEVs retained their colony forming potential (Figure 1D). This indicates that progenitors did not differentiate, but maintained their stemness. This notion was further supported by analyzing the presence of a more immature CD34^+^ subset (CD34^+^ CD38-CD45RA−, addressed here as primitive HSCs) and observed that the number of primitive HSCs was also maintained by both aEVs and fEVs treatment, comparable to the control culture condition (Figure 1E). Together, these findings demonstrate that aEVs support the *ex vivo* expansion of UCB-CD34^+^ cells while maintaining primitive HSCs in culture.

### Both Adult and Fetal BMSCs Release EVs With Similar Morphological Characteristics

**FIGURE 2:**
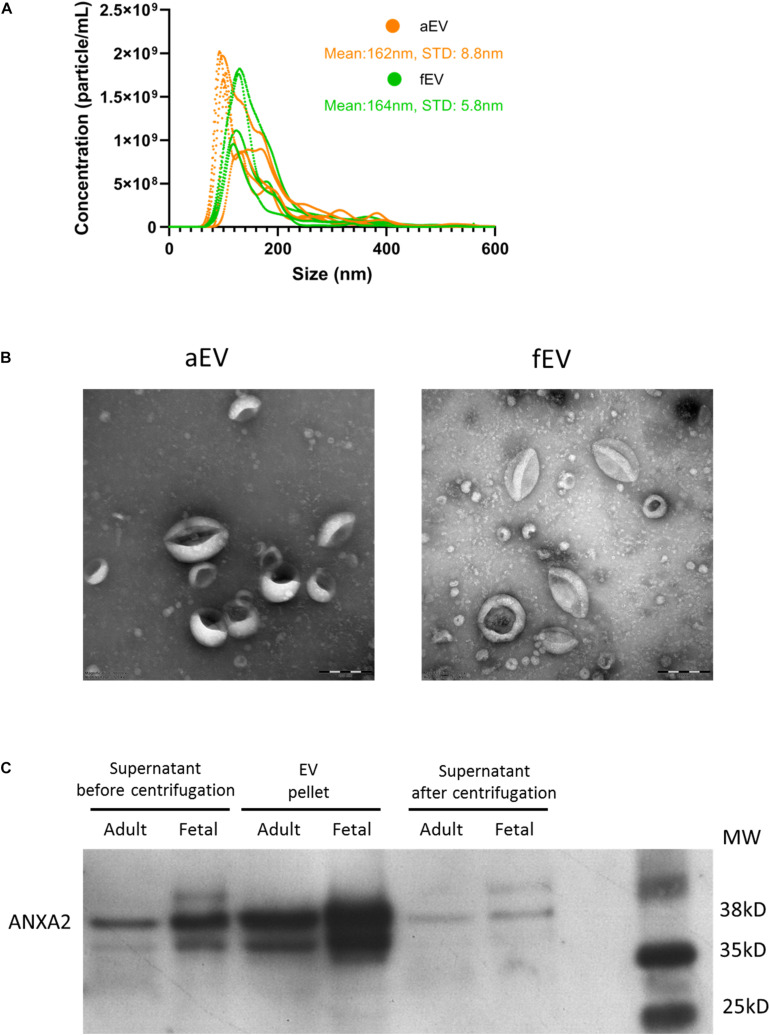
MSC derived EVs characterization. **(A)** EV size and concentration (particle/mL) were analyzed using nanoparticle tracking analysis. We used *n* = 5 aEV (*n* = 3 single and *n* = 2 pooled donors) and *n* = 4 fEV (single donors). The highest concentration of particles detected in aEVs and fEVs have the size of 100–200 nm, with a mean particle size of 162 and 164 nm, respectively. **(B)** Transmission electron microscopy images confirm the presence of different sized EVs, characterized by the cup-shaped morphology. Scale bar = 200 nm. **(C)** Western blot analysis of the MSC supernatant before centrifugation, EV pellet and supernatant after centrifugation (containing no EVs), and using Annexin A2 as a positive marker for EVs, confirms the enrichment of EVs in the EV pellet.

Next, we used various methods to characterize the EVs obtained from the different sets of BMSCs. Nanoparticle tracking analysis (NTA) of the isolated EVs indicated that the majority of detected particles were in the same size range of 100–200 nm, with no difference in mean size observed between aEVs (162 ± 9 nm) and fEVs (164 ± 6 nm) (Figure 2A). Transmission electron microscopy (TEM) analysis confirmed the presence of a heterogeneous EV population, with particles size comparable to the NTA-derived data. The isolated EVs displayed a cup shaped morphology that is characteristic for EVs analyzed by TEM (van Niel et al., 2018) and we observed no differences in morphology between aEVs and fEVs (Figure 2B). Western blot analysis using an EVs specific marker, Annexin A2, confirmed the enrichment of EVs in the 100,000 g EV pellet compared to supernatant before centrifugation, and the absence of Annexin A2 in the supernatant after centrifugation (Figure 2C). These findings, in line with the aforementioned functional assays, strengthen our hypothesis that BMSC secrete EVs with supportive factors for *ex vivo* expansion of the UCB-CD34^+^ cells.

### Protein Profiling of Adult and Fetal BMSC-Derived EVs Identifies TGFB1 as a Key Suppressor in the Expansion of UCB-CD34^+^ Cells by Fetal MSC Derived EVs

**FIGURE 3:**
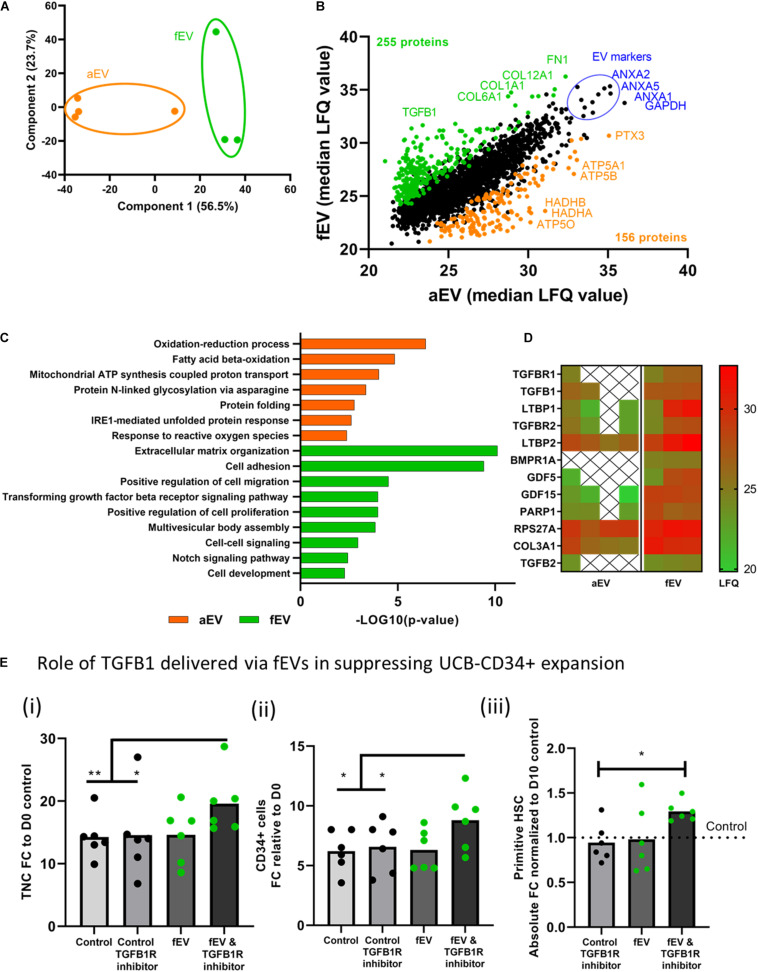
Proteomic profiling of adult- and fetal MSC derived EVs. **(A)** Principal Component Analyses, PCA, based on the LFQ intensity values of proteins that were quantified in all samples, showing a separation between aEVs and fEVs. Each datapoint represents one independent sample (*n* = 2 single and *n* = 2 pooled adult MSC EV donors and *n* = 3, single fetal MSC EV donors). **(B)** Scatterplot results of all quantified proteins, represented as median LFQ values after imputation, showing 156 differentially expressed proteins identified in aEVs (orange) vs. 255 differentially expressed proteins in fEVs (green). The top highly expressed proteins are representative EV protein markers (blue). **(C)** Gene Ontology enrichment analysis of the differentially expressed proteins based on the “Biological process.” **(D)** Heatmap showing significantly enriched fEV proteins involved in transforming growth factor receptor signaling pathway and potentially responsible for limiting the effect of fEVs to support the *ex vivo* expansion of UCB CD34^+^ cells. Each column represents one independent sample (X = not quantified). **(E)** Inhibition of TGFB1 signaling pathway in UCB-CD34^+^ cells. UCB-CD34^+^ cells (*n* = 6 donors) were cultured for 10 days in growth factor-driven serum-free expansion media in the presence or absence of fEVs (*n* = 6, single donors) and TGFB1R inhibitor; (i) proliferation of viable total nucleated cells, TNC and (ii) CD34^+^ cells shown as fold change increase compared to D0 input.; (iii) proliferation of primitive HSC (CD34^+^ CD38-CD45RA-) subset, shown as absolute fold change normalized to D10 control. Blocking TGFBR1 shows a non-significant trend in increased expansion of total nucleated cells (*P* = 0.14), the CD34^+^ cell subset (*P* = 0.11) and the primitive HSCs (*P* = 0.09) when compared to fEVs. *P < 0.05, **P < 0.01.

To assess whether the differences between aEVs and fEVs in supportive expansion of UCB-CD34^+^ cells can be attributed to their protein cargo, we performed label-free mass spectrometry-based proteomics analysis. We identified a total of 2,283 proteins that were detected in all biological replicates in at least one group (aEV or fEV) with 139 and 27 proteins quantified exclusively in fEV or aEVs, respectively. The proteins that were quantified in both subsets included 90 of the Top 100 most frequently identified exosomal proteins, as defined by the ExoCarta database (Keerthikumar et al., 2016; Supplementary Table 1). Principal component analysis with the identified proteins illustrated that the first two components account for 80.2% of the total variance and were able to separate the aEVs and fEVs on the first principal component (Figure 3A). Differential enrichment analysis indicated that 156 proteins were significantly enriched in aEVs (orange) and 255 proteins in fEVs (green) (Figure 3B). The top highly abundant proteins detected in both aEVs and fEVs are the well-defined EV marker proteins ANXA1, ANXA2, ANXA5, and GAPDH (blue) (Figure 3B). To further investigate the function of our differentially expressed proteins, we conducted Gene Ontology (GO) enrichment analysis using DAVID (Huang et al., 2009), and identified that the processes, such as oxidation-reduction process (GO:0055114; HADHA, HADHB), mitochondrial ATP synthesis coupled proton transport (GO:0042776; ATP5A1, ATP5B, and ATP5O) and protein folding (GO:0006457; FKBP11, FKBP10, FKBP2, MESDC2) were overrepresented in aEVs (Figure 3C). In contrast, the majority of the proteins overrepresented in fEVs were annotated to extracellular matrix organization (GO:0030198; FN1, COL12A1, COL1A1, COL6A1, CCDC80, and others), positive regulation of cell migration (GO:0030335; ITGA4, ITGA6, GDF15, SEMA3, APDSD6, MMP14, ICAM1, FGFR1, PDGFRA, ADAMTS1), and proteins involved the transforming growth factor beta receptor signaling pathway (GO:0007179; TGFBR1, TGFB1, LTBP1, TGFBR2, LTBP2, BMPR1A, GDF5, GDF15, PARP1, RPS27A, COL3A1, TGFB2; Figures 3C,D).

For the latter one, we used a heat map to visualize the detection intensities of the proteins involved in the transforming growth factor beta receptor signaling pathway and noticed that some of these proteins were limited or not quantified in the aEVs, whereas they were quite abundant in the fEVs (Figure 3D). Since TGFB1 was previously described in literature to negatively regulate the number and function of hematopoietic stem cells (Wang et al., 2018), we investigated whether TGFB1 transported by the fEVs is responsible for the suppression of the potentially supportive factors within the EV cargo. Therefore, we exposed UCB-CD34^+^ cells to fEVs in the absence or presence of transforming growth factor beta receptor 1 inhibitor (TGFBR1 inhibitor). We found that blocking TGFBR1 significantly increased the expansion of total nucleated cells, the CD34^+^ cell subset and the primitive HSCs when compared to control culture conditions (Figures 3Ei–iii). These results are compatible with the hypothesis that TGFB1 present in fEVs impairs their supportive effects on the HSC expansion, but it also indicates that both aEVs and fEVs contain supportive factors for *ex vivo* expansion of UCB-CD34^+^ cells. Therefore, we investigated the top 30 highly expressed proteins between aEVs and fEVs to identify candidates that may induce UCB-CD34^+^ cell expansion (Supplementary Figure 2). Among them, we identified well defined EV markers (blue), proteins involved in extracellular matrix organization (GO:0030198; green) and proteins involved in cell activation (GO: 0001775).

Together, we found various proteins (e.g., TGFB) that were differentially quantified between adult and fetal EV cargo, and suggests that aEVs contain a more favorable ratio of HPSC supportive protein cargo.

### Identification of Small RNA Expression Signature in EVs Derived From Adult and Fetal BMSCs

Besides the identified proteins transfered via the BMSC-derived EVs, the presence of a selective repertoire of small RNAs can additionally contribute to the *ex vivo* expansion of UCB-CD34^+^ cells. Small non-coding RNAs represent an interesting group of bioactive molecules that may be involved in reprogramming UCB-CD34^+^ cell fate (De Luca et al., 2016). To assess which small RNAs are selectively packed in the BMSC derived EVs and may be transferred to UCB-CD34^+^ cells, we performed next generation sequencing of aEVs and fEVs.

**FIGURE 4:**
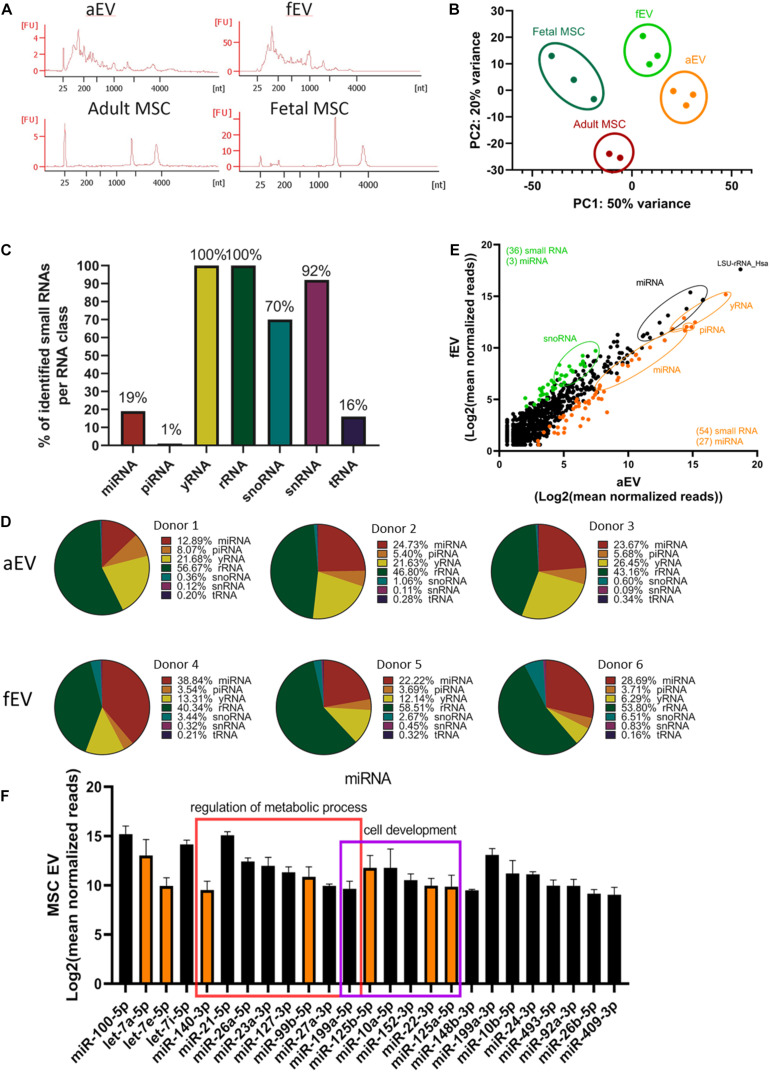
Small RNA profiling of adult and fetal MSC derived EVs. **(A)** Representative RNA profiles of aEVs (*n* = 1 single and *n* = 2 pooled donors) and fEVs (*n* = 3, single donors) compared to their parental cells. (FU, Fluorescent Units) **(B)** Principal component analyses of normalized small RNA sequencing data showing a nice separation between the different biological replicates. **(C)** Percentages of identified small RNAs per RNA class. **(D)** Pie charts mapping the composition in percentage of the diverse small RNA species, per each biological replicate. For each EV sample we individually summed the number of reads per RNA species, and presented the results in percentages in form of pie-charts **(E)** Scatterplot mapping Log2 (mean normalized reads) of aEVs (differentially expressed in orange) and fEVs (differentially expressed in green). **(F)** Common most abundant miRNAs identified in aEVs and fEVs with Log2 (mean normalized reads) higher than nine. Some of the miRNAs were significantly expressed in aEVs (orange); some miRNAs were annotated to regulation of metabolic process (red square) and cell morphogenesis or development (purple square). Error bars represent the standard deviation of the normalized reads from all EVs (3× fEVs and 3× aEVs) combined.

Analysis of the total RNA from both adult and fetal MSC-derived EVs revealed a typical RNA size distribution profile of vesicles, which were enriched for small RNAs and highly reduced in the 18S and 28S rRNA peaks, when compared to their parental cells (Figure 4A). Principal component analysis of small RNA sequencing depicted a separation between the vesicular and cellular small RNAs, and between the adult and fetal origin (Figure 4B). To identify the small RNA distribution biotypes, we mapped all detected RNA reads to known small RNA sequences and determined that 19% of the miRNA, 1% of the piRNA, 100% of the yRNA, and rRNA, 70% of the snoRNA, 92% of the snRNA, and 16% of the tRNA, were present in our EV samples (Figure 4C). Next, we determined whether there are differences between aEVs and fEVs in the number of reads identified per RNA species (Figures 4D,E). We found that EV samples were more abundant in rRNA (49.9% ± 7.5), miRNA (25.2 ± 8.5%) and yRNA (16.9 ± 7.5%), while snoRNA was mainly only identified in fEVs (4.2 ± 2%) and limited in aEVs (0.7 ± 0.4%). While little is known about the contribution of this different small RNA species to the hematopoietic processes, miRNAs have been lately described to play a key role in the hematopoietic system, being involved in the maintenance of self-renewal of hematopoietic stem cells and differentiation into mature blood cells. Moreover, miRNA are highly abundant in aEVs and fEVs and encouraged us to search for candidate miRNAs that may drive UCB-CD34^+^ cell expansion. Hence, we outlined all miRNAs that have a Log2 (mean normalized reads) count, over all adult, and fetal samples, higher than nine (Figure 4F) and used miRPathDB to determine the top GO biological processes associated with the listed miRNAs (Kehl et al., 2020). Target genes of the miRNAs were mostly annotated to regulation of metabolic process (miR-21-5p, miR-26a-5p, miR-23a-3p, miR-125b-5p, miR-127-3p, miR-99b-5p, miR-27a-3p, miR-199a-5p) and cell morphogenesis or development (miR-125b-5p, miR-10a-5p, miR-152-3p, miR-22-3p, miR-125a-5p, miR-148b-3p). Among these miRNAs, we identified interesting miRNA clusters, such as miR-99b, let-7e, and miR-125a or miR-99a/100, let-7, and miR-125b, which were previously found to increase the number of HSCs *in vivo* (Guo et al., 2010; Emmrich et al., 2014), making them potential candidates for the induction of *ex vivo* UCB-CD34^+^ cell expansion.

### EVs Derived From Adult and Fetal BMSCs Result in a Unique Transcriptional Response in UCB-CD34^+^ Cells

The proteomics and small RNA sequencing analyses of the EV cargo resulted in many targets that may be responsible or partly responsible for the supportive effects of the aEVs. To analyze what functional changes occur upon addition of aEV and fEVs, we determined the gene expression changes after 24 h vesicle incubation with UCB-CD34^+^ cells and compared this with the cells treated with cytokine only.

**FIGURE 5:**
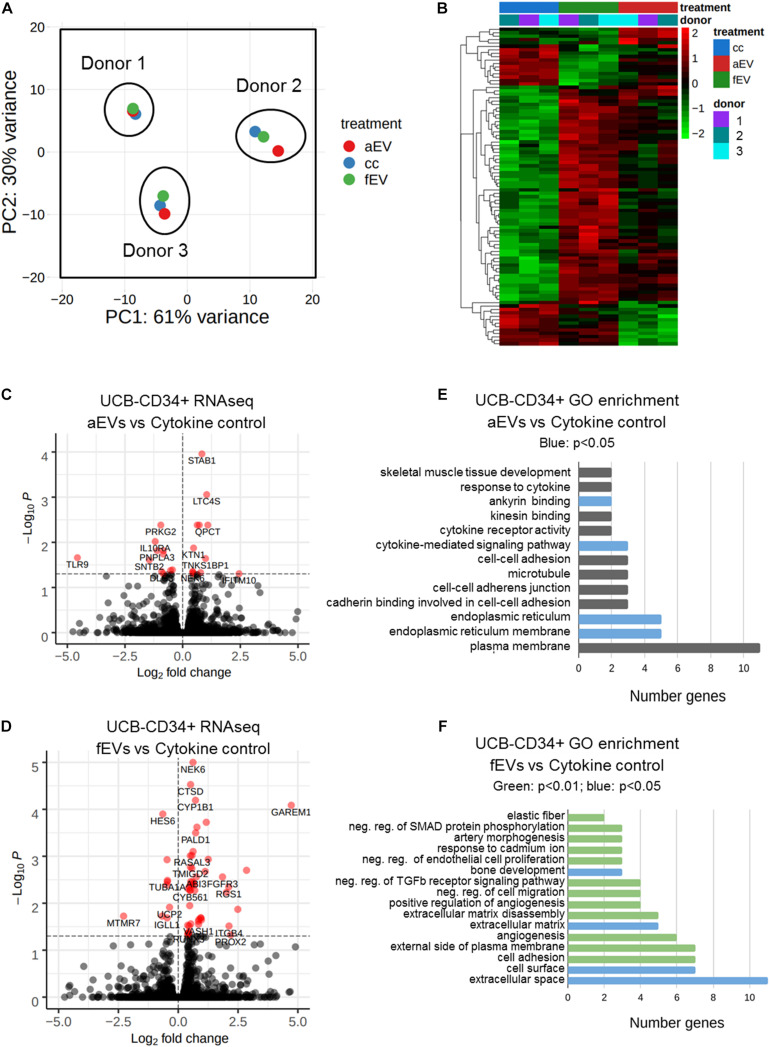
Differential gene expression analyses of UBC-CD34^+^ cells upon exposure to adult and fetal MSC EVs. **(A)** Principal component analysis of RNA sequencing data of UCB-CD34^+^ (*n* = 3) upon exposure for 24 h to cytokine cocktail with or without aEVs (*n* = 3, pooled donors) or fEVs (*n* = 3, single donors). **(B)** Heatmap of all differential expressed genes (p.adj < 0.05) in all comparisons aEV vs. cytokine control (cc), fEV vs. cc and aEV vs. fEV. **(C)** Volcano plot of UCB-CD34^+^ cells treated with aEVs vs. cc. **(D)** Volcano plot of UCB-CD34^+^ cells treated with fEVs vs. cc. **(E)** GO enrichment analyses using DAVID of all genes differentially regulated of UCB-CD34^+^ cells treated with aEVs vs. cc. Blue bars represent significant (*p* < 0.05) GO-term enrichment **(F)** GO enrichment analyses using DAVID of all genes differentially regulated of UCB-CD34^+^ treated with fEVs vs. cc. Blue and Green (*p* < 0.01) represent significant (*p* < 0.05 and *p* < 0.01, respectively) GO-term enrichment.

Our results indicated that the variation between the UCB-CD34^+^ cells from the different donors was larger than the differences of the treatment (Figure 5A). However, gene expression analyses indicated 93 genes that were differentially expressed between the various conditions and only 10 genes that were differentially expressed when cells were treated with fEVs compared to aEVs. Of these, we identified 5 genes, (e.g., MTF1, PER1, HOMER1, HSPA6, ENSG00000260534) that were significantly upregulated in response to aEV compared to fEV treatment of UCB-CD34^+^ cells (Figure 5B and Supplementary Table 2). Moreover we identified that 31 and 75 genes were differentially expressed (*p* < 0.05) upon aEV and fEV treatment, respectively, when compared to the UCB-CD34^+^ cells that were treated with cytokines only (Figures 5B–D).

Functional analysis of the 31 differentially expressed genes upon treatment with aEVs indicated that gene ontology terms such as endoplasmatic reticulum (GO:0005783), cytokine-mediated signaling pathway (GO:0019221) or involvement in ankyrin binding (GO:0030506) were enriched (Figure 5E). Interestingly, among the 75 genes differentially expressed upon addition of fEVs we identified GO categories, such as cell adhesion (GO:0007155), extracellular matrix (GO:0031012) and negative regulation of transforming growth factor beta receptor signaling pathway (GO:0030512) (Figure 5F).

In summary, our results on protein and miRNA cargo analyses of the EVs together with the gene analysis of the CD34^+^ cells suggests that especially the TGFB pathway hampers the effect of the fEVs and that the aEVs contain specific miRNA clusters that favor HSPC expansion.

## Discussion

In the current study, we illustrate that primary human BMSC-derived EVs isolated from fetal and adult bone marrow sources have a different supportive role on UCB-CD34^+^ cell expansion. We systematically investigated the bioactive cargo released by aEVs and fEVs, i.e., proteins and small RNA, and identified potential regulators that may have a positive supportive role on the *ex vivo* expansion of UBC-CD34^+^ cells while retaining CFU-GM capacity.

Among the bioactive molecules that we identified, small non-coding RNAs may represent as key regulators of UCB-CD34^+^ cell fate. Previous work of us and others have already illustrated that small non-coding RNAs are present in EVs that were isolated from osteoprogenitors and osteoblasts (De Luca et al., 2016; Morhayim et al., 2016, 2020; Xie et al., 2016). Here, we show that aEVs and fEVs contain many small RNAs with different abundances. While aEVs are abundant for small non-coding RNAs of the categories: yRNA and piRNA; snoRNAs were more present in fEVs. This illustrates the large differences found in small RNAs identified in body fluids (Godoy et al., 2018). Especially of interest is the microRNA cluster containing miR-99b, let-7e, and miR-125a that was higher enriched in the aEVs and within the Top50 most abundant miRNAs. miR-125a controls the size of the stem cell population by regulating hematopoietic stem/progenitor cell apoptosis (Guo et al., 2010; Wojtowicz et al., 2019). Although downregulation of proapoptotic genes were not identified in the RNAseq analyses of UCB-CD34^+^ cells in response to aEVs and fEVs, inhibition of apoptosis could take place at later stage or only present in subset of expanding UCB-CD34^+^ cells. In more recent work by Wojtowicz et al. (2019), miR-125a was identified to expand murine long-term repopulating hematopoietic stem cells and increase the number of hematopoietic stem cells *in vivo*. In addition, similar to the study of Xie et al., we identified miR-21 that has been reported to be involved in hematopoiesis (Bhagat et al., 2013; Xie et al., 2016). Altogether, we demonstrate that EVs contain molecules that may be directly involved in the supportive action and recent data suggests that miR-125a is sufficient to increase *ex vivo* of HSPCs.

Another interesting finding is the identification of proteins associated with TGF-β signaling and the regulation of negative regulators of TGF-β in UCB-CD34^+^ in response to fEVs. The low abundance of proteins involved in TGF-β signaling in aEVs let us further investigate whether the presence of TGFβ signaling is inhibitory in the fEVs assisted expansion conditions. Previously we showed that Transforming Growth Factor Beta Induced (*TGFBI*) expression in the bone marrow niche is essential for a balanced HSPC proliferation and differentiation, and only in the presence of BMSCs, TGFBI levels were reduced in HSPCs enhanced HSC maintenance (Klamer et al., 2018). This correlates very well with the reduced abundance of *TGF-*β in aEVs and their increased supportive effects. Nevertheless, several reports illustrated that TGF-β1 results in a biphasic dose-dependent response in HSC (Vaidya and Kale, 2015). Low concentrations of TGF-β1 are able to induce p44/42 MAPK-STAT pathway whereas high concentrations result in induced SMAD3 pathway activation and proliferation. In addition, TGF-β1 regulates distinct HSC subtypes. Early HSPC seem to be more sensitive to the inhibition by TGF-β1 while more differentiated progenitors get stimulated. Moreover, we also observed that genes involved in the negative regulation of TGFβ signaling were affected upon fEVs addition. Although we only identify 4 genes (*SKIL*, *SMAD7*, *LDLRAD4*, *ENG*), it is interesting to speculate that fEVs induce a TGF-β response with reduced *ex vivo* HSPC support. Inhibition of TGFBR by a neutralizing antibody prior to fEV addition indicated that an increased *ex vivo* expansion of UCB-CD34^+^ is obtained and suggests additional mode of action.

The question remains whether our findings could eventually lead to an innovative EV component-based GMP-compliant approach to *ex vivo* expand UCB-derived HSCs for therapeutic purposes. In previous years various clinical studies were initiated in which UCB derived CD34^+^ cells were expanded *ex vivo* using several factors, like StemRegenin 1, UM 171, TEPA, immobilized notch ligand Delta1 and nicotinamide (Lima et al., 2008; Delaney et al., 2010; Fares et al., 2014, 2017; Horwitz et al., 2014; Wagner et al., 2016; Stiff et al., 2018; Cohen et al., 2020). All components have shown to enhance HSC self-renewal and/or inhibit differentiation, and were suggested to improve *ex vivo* expansion of (primitive) HSCs. In all previous studies the HSPC support was established by the use of the specific component, as aEVs exerted their effect in this study, in combination with a cocktail of cytokines. However, the cytokine combinations used in the above-mentioned clinical studies were in general more extensive than our cocktail consisting of only two cytokines, SCF and Flt3L. Therefore it is difficult to compare the *in vitro* enhancing effects of the various compounds with our approach. In general, these clinical studies revealed that a higher total cell number (TNC) was closely associated with faster neutrophil engraftment in transplanted patients compared to historical controls, while in some cases platelet recovery was also statistically improved. Furthermore, only the studies using nicotinamide, UM171 and StemRegenin 1 reported long-term chimerism after transplantation of the expanded UCB-unit. These studies suggest that there is still much room for improvement for patient outcome and that the *in vivo* function of EVs and their role in hematopoietic support has to be further studied.

In conclusion, our study gives novel insights into the complex biological role of EVs in the bone marrow microenvironment. Systematic analyses of supportive and less supportive primary BMSC derived extracellular vesicles indicated known molecules, e.g., microRNA cluster miR-99b/let-7e/miR-125a, and the presence of TGFb pathway components that are important regulators in the cell-cell communication via EVs and open new means for the application of EVs in the discovery of therapeutics for more efficient *ex vivo* HSPC expansion.

## Data Availability Statement

The datasets presented in this study can be found in online repositories. The names of the repository/repositories and accession number(s) can be found in the article/Supplementary Material.

## Author Contributions

CG, JM, EBr, BE, CV, and JP contributed to the conception and design of the experiments. CG, MKl, and MKo isolated EVs, perform *ex vivo* expansion experiment, flow cytometry experiments, and data analyses. CG, FA, and MB performed mass spectrometry analyses. EBi and RH performed smallRNA and RNAseq experiments. JP analyzed smallRNA and CD34 RNAseq data. CG performed statistical data analyses, generated the figures, and wrote the first versions of the manuscript, supervised by CV and JP. CG, JM, MKl, MKo, SE, RH, EBi, FA, MB, MN, BE, EBr, CV, and JP contributed to manuscript revisions, read, and approved the submitted version. All authors contributed to the article and approved the submitted version.

## Conflict of Interest

The authors declare that the research was conducted in the absence of any commercial or financial relationships that could be construed as a potential conflict of interest.

## Footnotes

1 https://www.ncbi.nlm.nih.gov/geo/

2 http://proteomecentral.proteomexchange.org

## Supplementary Material

Supplementary Figure 1 **(A)** Concentration dependent effect of fEVs on TNC proliferation of UCB CD34^+^ cells. Different UCB-CD34^+^ cell donors (*n* = 3) co-cultures with EVs derived from different single donors (*n* = 3), **(B)** aEVs (single donors, *n* = 2), but not serum free MSC medium or supernatant after EVs isolation, show a trend in positive contribution to the proliferation of UCB CD34^+^ cells over a period of 10 days.

Supplementary Figure 2 Top 30 most abundant proteins among both aEVs and fEVs displayed according to their expression level (mean ± SD), colors are in accordance with previous description.

Supplementary Table 1 Proteomics data. Proteomics identification of all proteins present in aEVs and fEVs (*n* = 3 each). Proteins identified in both (1) aEVs and fEVS (2283 proteins), proteins that were identified only in aEVs (2) or fEVs (3), proteins that were overrepresented in aEVs (4), or in fEVs (5), comparison of the top100 Exocarta (6) proteins identified in fEVs and aEVs.

Supplementary Table 2 small RNAseq data. Result output of the differential gene expression analyses of small RNA sequencing content of adult cells (AMSC, *n* = 2), adult EVs (AMSC.EV, *n* = 3), fetal Cells (FMSC, *n* = 3 donors), fetal EVs (FMSC.EV, *n* = 3 donors). Result output after differential gene expression analyses with DESeq2 (Love et al., 2014).

Supplementary Table 3 Differential gene expression analyses of human UCB-CD34^+^ 24 h incubated with aEVs, fEVs, and cytokine control. Result output of the differential gene expression analyses in UCB-CD34^+^ incubated for 24 h with aEVs, fEVs or cytokine control (CC) (*n* = 3 per treatment). Result output after differential gene expression analyses with DESeq2 (Love et al., 2014).
