# Supplementary figures and images for: Extracellular Vesicles Derived From Adult and Fetal Bone Marrow Mesenchymal Stromal Cells Differentially Promote ex vivo Expansion of Hematopoietic Stem and Progenitor Cells
Source: Front Bioeng Biotechnol. 2021 Feb 25;9:640419. doi: 10.3389/fbioe.2021.640419 (PMC7947881; doi:10.3389/fbioe.2021.640419)

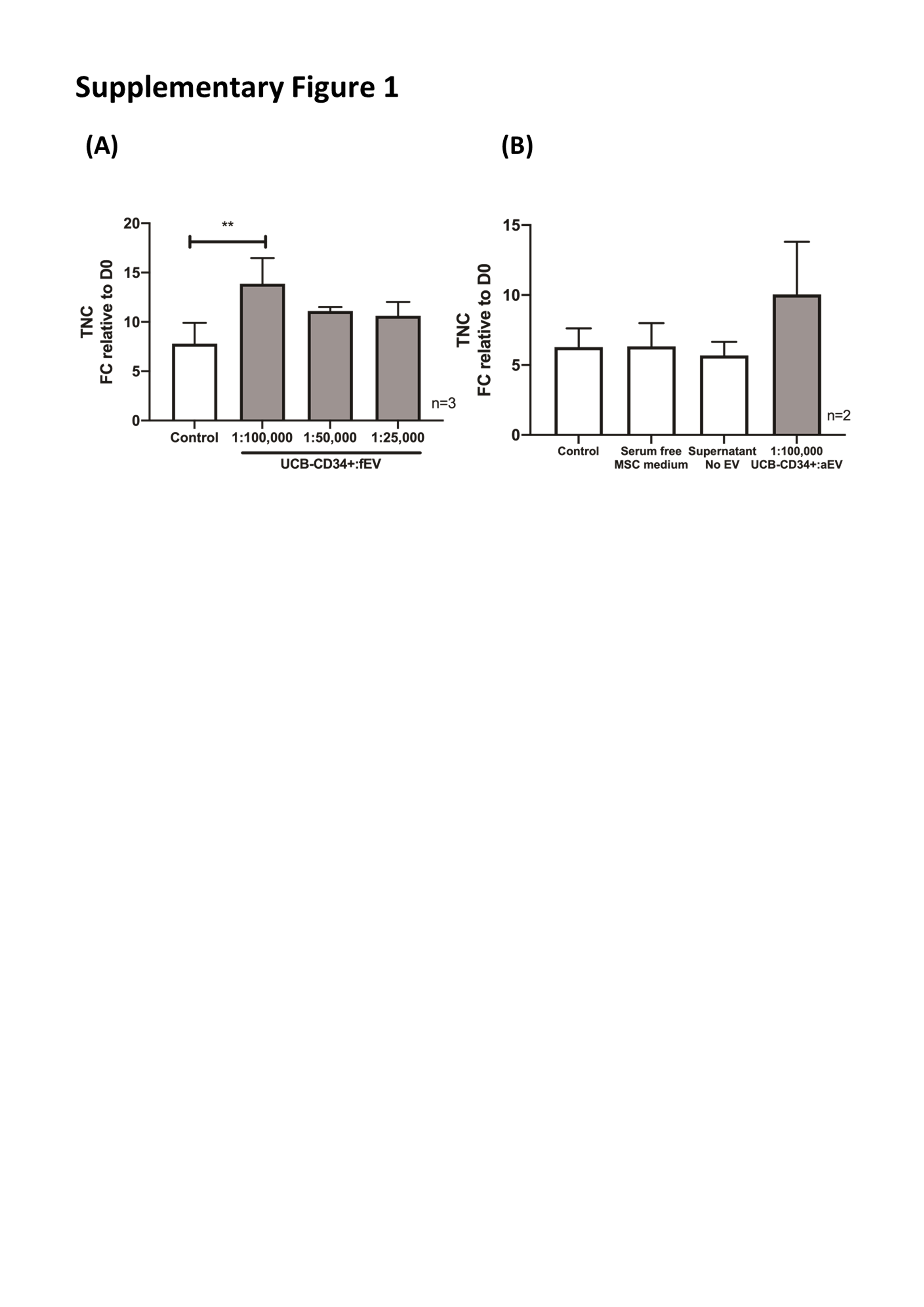

Supplement: Supplementary Figure 1 — (A) Concentration dependent effect of fEVs on TNC proliferation of UCB CD34+ cells. Different UCB-CD34+ cell donors (n = 3) co-cultures with EVs derived from different single donors (n = 3), (B) aEVs (single donors, n = 2), but not serum free MSC medium or supernatant after EVs isolation, show a trend in positive contribution to the proliferation of UCB CD34+ cells over a period of 10 days. [file Image_1.TIF]

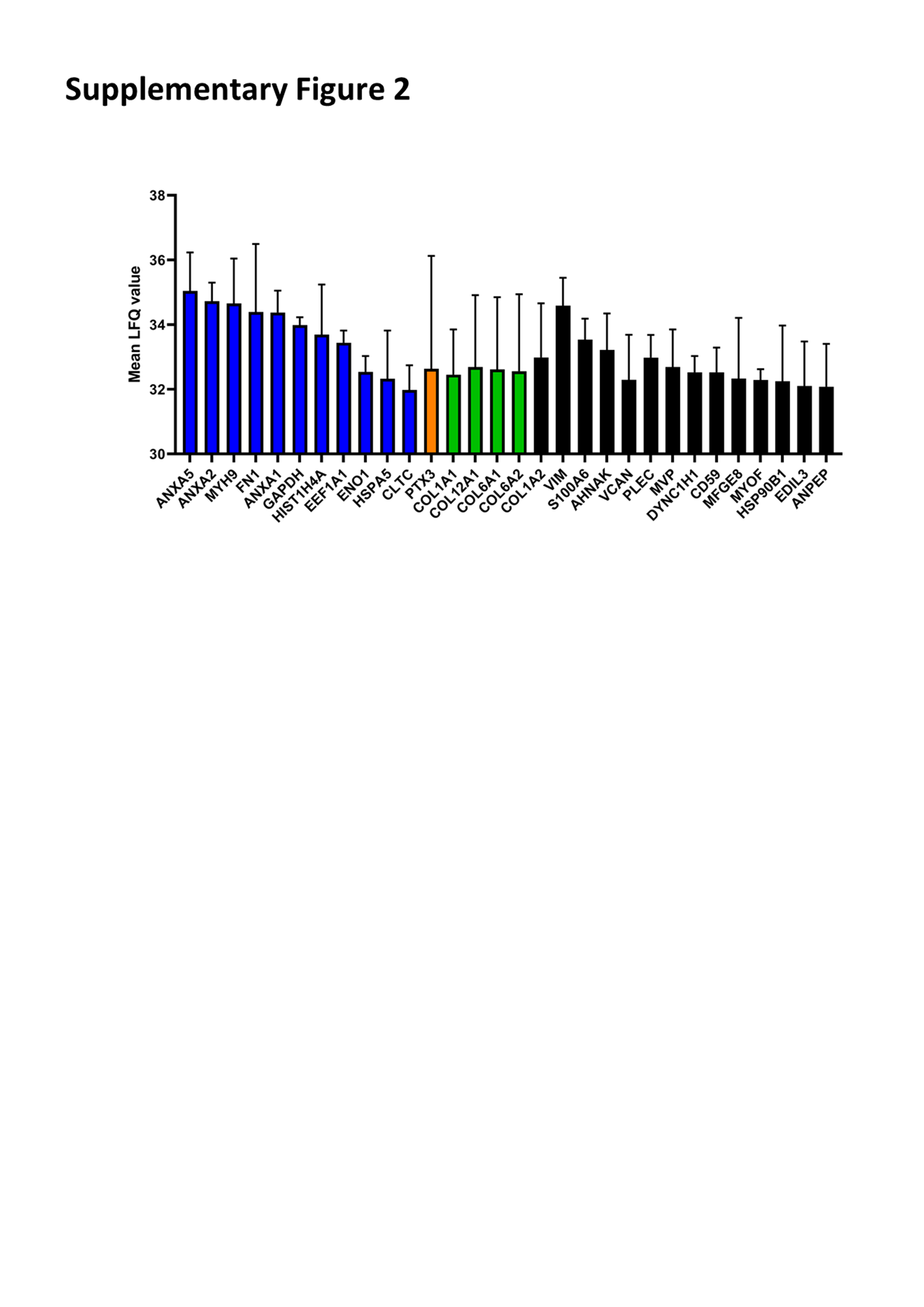

Supplement: Supplementary Figure 2 — Top 30 most abundant proteins among both aEVs and fEVs displayed according to their expression level (mean ± SD), colors are in accordance with previous description. [file Image_2.TIF]
